# Clinical characteristics and factors associated with mucus plugs under bronchoscopy in children hospitalized for acute asthma attack

**DOI:** 10.3389/fped.2024.1382680

**Published:** 2024-10-14

**Authors:** Peng Han, Anxia Jiao, Ju Yin, Huimin Zou, Yuliang Liu, Zheng Li, Quan Wang, Jie Wu, Kunling Shen

**Affiliations:** ^1^ Respiratory Department, Beijing Children’s Hospital, China National Clinical Research Center of Respiratory Diseases, National Center for Children’s Health, Capital Medical University, Beijing, China; ^2^ Department of Interventional Pulmonology, Beijing Children’s Hospital, National Center for Children’s Health, Capital Medical University, Beijing, China; ^3^ Pediatric Intensive Care Unit, Beijing Children’s Hospital, National Center for Children’s Health, Capital Medical University, Beijing, China; ^4^ Department of Emergency, Beijing Children’s Hospital, National Center for Children’s Health, Capital Medical University, Beijing, China; ^5^ Department of Respiratory, Shenzhen Children’s Hospital, Shenzhen, China

**Keywords:** mucus plugs, asthma attack, children, bronchoscope, factors

## Abstract

**Objective:**

To describe clinical characteristics of hospitalized children with acute asthma attacks complicated with mucus plugs and to investigate the factors associated with mucus plugs in asthma children.

**Methods:**

This retrospective study analyzed hospitalized children and adolescents with acute asthma attacks from January 2016 to December 2021. The demographic information and characteristics were collected. Subjects were categorized into the mucus plug group and the control group based on the bronchoalveolar lavage results. The Logistic regression analyses were utilized to assess the relative factors associated with mucus plugs. All data were analyzed using SPSS 22.0.

**Results:**

This study included 242 individuals. Out of the 151 subjects who underwent bronchoscopy, 62.9% were classified in the mucus plug group and 37.1% in the control group. The subjects with dyspnea had a higher proportion in the mucus plug group (52.6% vs. 26.8%). The serum total IgE level of the mucus plug group was lower than the control group. The proportion of subjects who were diagnosed with asthma for the first time during hospitalization (87.4% vs. 76.8%) and combined with respiratory infection (91.6% vs. 82.1%) in the mucus plug group might be higher than that in the control group. More subjects in the mucus plug group were administered systemic glucocorticoid, magnesium sulfate, aminophylline, and mucolytic drugs after hospitalization. In multivariable analysis, diagnosed with asthma for the first time during hospitalization (OR = 4.404; 1.101–17.614), dyspnea (OR = 4.039; 1.306–12.496), and cesarean (OR = 0.274; 0.092–0.812) might be associated with mucus plug in children hospitalized for an acute asthma attack.

**Suggests:**

While our retrospective study suggests that some clinical features of children hospitalized with asthma who have mucus plugs differ from those without, further studies are required.

## 1 Introduction

Mucus hypersecretion is one of the pathophysiological features of asthma (1). During acute asthma attacks, restricted airflow and weakened coughing lead to decreased mucus discharge, which causes an increase in secretion accumulation. This buildup results in mucus plugging that obstructs the airway. For over a century, researchers have studied the formation of mucus plugs in the airways of asthma. In 1922, Huber HL discovered the presence of extensive mucus plugs in the airways of deceased patients with asthma (2). Similarly, in 1959, Dunnill made the same discovery (3). Asthma children with mucus plugs have a higher risk of acute asthma attacks and poor response to treatment.

Since the initial publication of the first diagnostic report on children's fiberoptic bronchoscopy in 1978, bronchoscopy has emerged as a significant diagnostic and therapeutic tool for pediatric acute and chronic lung illnesses (4). Additionally, a bronchoscope operation can find and remove mucus plugs directly from the airway during examination, promptly alleviating symptoms. Currently, the lack of methods to quantify airway mucus has hindered research on the relative factors of mucus plugs in pediatric asthma patients. Therefore, our study focused on hospitalized asthma children whose bronchoscopy had been operated on, the proportion and clinical characteristics of mucus plugs in acute asthma attacks, and factors associated with mucus plugs.

## 2 Method

### 2.1 Subjects

The retrospective study based on an electronic medical record system (EMRS) to describe the clinical characteristics and assess the factors associated with mucus plugs was performed in children and adolescents with asthma. The inclusion criteria for the study were age between 3 and 18 years; and admission to Beijing Children's Hospital affiliated with Capital Medical University, a tertiary hospital also known as the National Children's Medical Center, for acute asthma attacks from January 2016 to December 2021.

The diagnosis of asthma and acute asthma attacks was based on the criteria of “Guidelines for Diagnosis and Prevention of Bronchial Asthma in Children (2016 Edition)” (5). The diagnostic criteria for asthma were described as a history of typical variable respiratory symptoms (including wheezing, shortness of breath, chest tightness, and cough), and confirmed variable expiratory airflow limitation (documented excessive variability in lung function and documented expiratory airflow limitation) (5). The definition of an acute asthma attack is the sudden onset of symptoms such as wheezing, coughing, shortness of breath, and chest tightness, or the sudden worsening of pre-existing symptoms. The choice for performing a bronchoscopy examination was based on the physician's professional opinion after clinical consultation and physical examination. The subjects were selected from the EMRS based on their discharge diagnosis, which included bronchial asthma, namely cases coded as J45.0–J45.9 and J46 in the International Statistical Classification of Diseases and Related Health Problems 10 (ICD-10). In cases where the chief complaint of physicians included wheezing, we classified these patients' admission reasons as acute attacks. Exclusion criteria included incomplete demographic information; lack of information on diagnosis and treatment after admission; hospital stay ≤1 day; with underlying diseases such as primary or secondary immunodeficiency disease, hereditary metabolic disease, tumor, and organ or hematopoietic stem cell transplantation; suffering from active pulmonary tuberculosis and with a history of asthma but hospitalized for other reasons.

### 2.2 Data collection

A database was created to record the variables to be obtained (Microsoft Excel Software, USA, 2010). A consecutive numerical value was assigned to each patient, and with this coding, they were entered into the database. In this way, the information recorded was completely anonymous and did not contain any value that allowed the study participants to be identified or traceability carried out. The information on demographics (such as age, gender, birth weight, etc.), pre-admission status (such as allergy history, asthma hospitalization history, history of systemic glucocorticoid therapy, etc.), respiratory infection, comorbidities, and disease characteristics (such as asthma phenotype, results of serum allergen detection and whole blood cell analysis, etc.) were collected using a pre-designed case report form. In our study, the duration of the IgE and allergen detection procedures was limited to a maximum of three months before the bronchoalveolar lavage procedure. However, the duration of procalcitonin, EOS, and CRP was limited to a maximum of one week before the bronchoalveolar lavage procedure.

### 2.3 Criteria for grouping

Based on the results of the bronchoalveolar lavage, individuals who exhibited strip or strip flocculent secretions were classified as the mucus plug group, while those with little to no secretion were classified as the control group. In our study, we adhered to a unified and standardized bronchoscopy process for all individuals. Based on age, they were divided into four groups: infants (<3 years old), preschoolers (3–5 years old), school-aged children (6–9 years old), and adolescents (≥10 years old). The participants were also categorized by the season of admission (spring, summer, autumn, and winter).

The severity classification was based on the criteria set by the Chinese Guidelines, which classified acute asthma attacks as mild, moderate, severe, or critical for children aged ≥6 years (as shown in Supplementary Table S1), and mild or severe for children aged <6 years (as shown in Supplementary Table S2) (5). In clinical practice, the diagnostic criteria for children's respiratory failure include the presence of an underlying condition causing respiratory failure; varying degrees of difficulty breathing and cyanosis; artery blood gas analysis revealing a PaO2 <60 mm of mercury (mmHg; 1 mmHg = 0.133 kPa) and/or PaCO2 >50 mmHg. The presence of dyspnea in subjects is based on observable signs such as respiratory rate, cyanosis, and breathing with accessory muscles occurring.

The comorbidities, which included rhinitis, sinusitis, nasal polyps, atopic dermatitis, food allergy, obesity, depression and anxiety, and ABPA, were evaluated based on the doctor's diagnosis and medical history information provided by the parents or the child himself. Based on the presence of type 2 (T2) inflammation, the phenotypes of asthma airway inflammation were classified into four categories: atopy-only (with blood eosinophils <470/µL, the sum of any sIgE ≥0.7 ku/L), Eos-only (with blood eosinophils ≥470/µL and all sIgE <0. 7 ku/L), T2-high (with blood eosinophils ≥470/µL and any sIgE ≥0.7 ku/L), or T2-low (with blood eosinophils <470/µL and all sIgE <0.7 ku/L) (6).

### 2.4 Ethics approval and research registration

Informed consent had been obtained from parents before performing flexible bronchoscope procedures for clinical purposes. The subjects cannot be found, and the research project did not involve personal privacy or commercial interests. Our study has received approval from the Ethics Committee to exempt participants from signing informed consent forms. This research protocol was approved by the Ethics Committee of Beijing Children's Hospital affiliated with Capital Medical University [the ethical approval number: (2023)-E-078-Y]. The study was registered at clinicaltrials.gov (NCT05800379) on 05/04/2023.

## 3 Statistical analyses

Descriptive analyses including means and standard deviations for numerical variables and percentages for categorical variables were used to analyze the main characteristics of the study sample. We compared the differences between the individuals with and without bronchoscopy examination and evaluated the heterogeneousness between the mucus plug group and the control group. The numerical variables were compared using the student's *t*-test or the Mann-Whitney test and the categorical variables using the chi-square or Fisher's exact test. By comparing the general data and clinical characteristics of subjects between the groups, the variables with differences were obtained (*P* < 0.2). Then we adopt binary stepwise backward multivariate Logistic regression analysis to study the relationship between multiple variables and results. The odds ratio (OR) and its 95% confidence interval (95% CI) were considered as a measure of association. The data were analyzed using SPSS 22.0, and significance was considered at *p* < 0.05.

## 4 Results

### 4.1 General information on enrolled subjects

**Figure 1:**
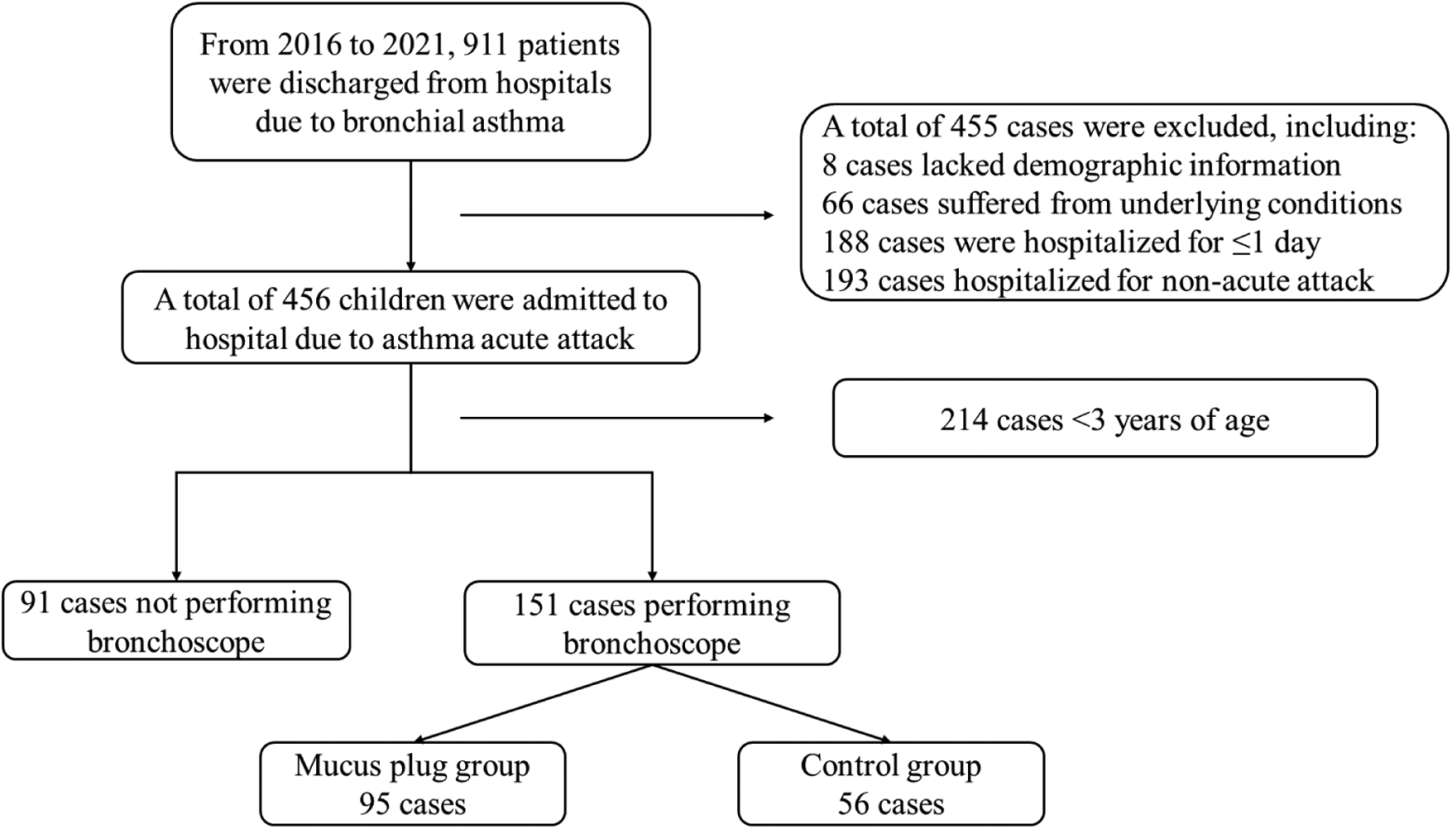
Enrolment and exclusion of subjects. This depicts the main workflow of the enrolment and exclusion of subjects. A total of 911 subjects had the diagnosis of asthma. Among them, 455 subjects met exclusion criteria, including 8 subjects who lacked demographic information; 66 subjects suffered from underlying conditions; 188 subjects were hospitalized for ≤1 day; 193 subjects were hospitalized for non-acute attacks. Among 456 children who were admitted to the hospital due to asthma attacks, 214 subjects were aged <3 years and 91 subjects did not perform bronchoscopes. Out of the 151 subjects who underwent bronchoscopy, 95 were classified in the mucus plug group and 56 in the control group.

**Figure 2:**
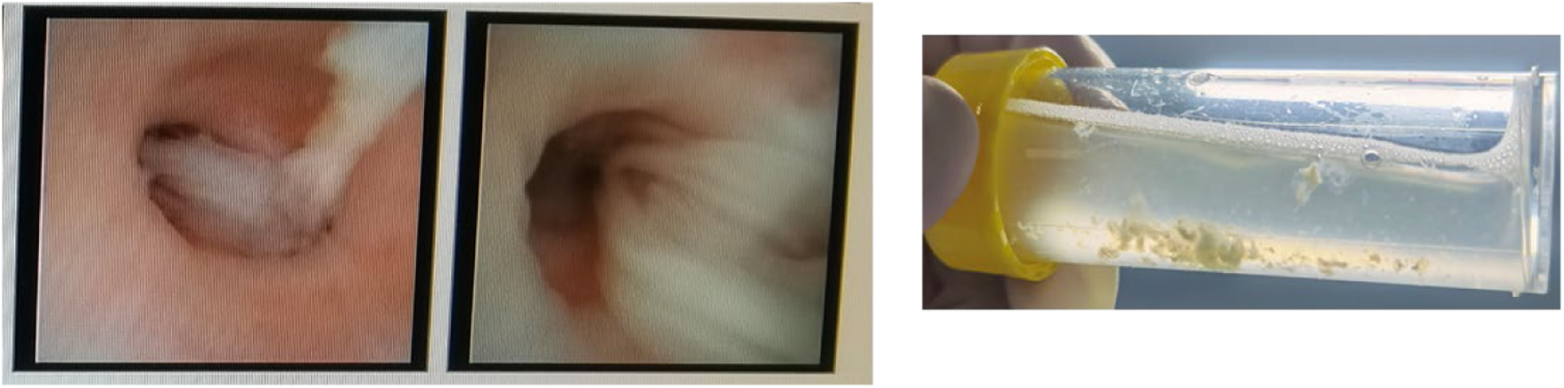
One representative bronchoscopy image of mucus plugs in asthma children. The subject was a male, aged four years and three months. The patient presented with a chief complaint of a cough that had persisted for three days, accompanied by wheezing and a fever that occurred once daily.

In the EMRS, 911 subjects were diagnosed with asthma. A total of 242 subjects participated in this study, as illustrated in Figure 1. Among the subjects, 62.9% (95/151) of the cases were classified into the mucus plug group and 37.1% (56/151) in the control group. The representative image of mucus plugs in asthma children under bronchoscopy was shown in Figure 2. 143 male subjects (59.1%) and 99 female subjects (40.9%) were included in the study, with an age range of 3.00–17.08 years.

When comparing the general characteristics of subjects who underwent bronchoscopy examination to those who did not, we found no significant differences except for a history of asthma hospitalization. The percentage of subjects who had been hospitalized for asthma within the past 12 months was higher in the group that underwent bronchoscopy compared to those who did not (21.5% vs. 7.7%, *P* = 0.005; as shown in Supplementary Table S3). The percentage of subjects who were admitted to the hospital through the emergency department (51.7% vs. 75.8%) and required treatment in PICU (17.2% vs. 26.4%) were lower in the group that underwent bronchoscopy compared to those who did not (*P* < 0.05; as shown in Supplementary Table S4). Although a statistical difference in procalcitonin, white blood cell counts, neutrophil count, eosinophil count, and percentage of eosinophil was found between the two groups, this difference was not clinically significant in a clinical setting (as shown in Supplementary Table S4).

### 4.2 Comparison of clinical data between the mucus plug group and the control group

There were no significant differences in age (6.83 years vs.6.13 years), gender (male, 60.0% vs. 55.4%), and type of residence (urban living, 74.7% vs. 78.6%) between the two groups (*P* > 0.05). There were neither statistically significant differences in birth weight (3.30 kg vs. 3.25 kg), premature infants (4.4% vs. 9.3%), cesarean (44.7% vs. 57.4%), neonatal asphyxia (0.0% vs. 1.9%), neonatal oxygen (1.1% vs. 1.9%), feeding mode (breastfeeding, 75.5% vs. 72.7%; formula feeding, 13.8% vs. 7.3% and mixed feeding, 10.6% vs. 20.0%), allergy history (38.9% vs. 46.4%), allergic family history (41.5% vs. 20.0%), asthma hospitalization history (25.8% vs. 14.3%), and systemic glucocorticoid therapy(14.0% vs. 7.3%) between the mucus plug group and the control group (as shown in Table 1, all *P* > 0.05).

**Table 1:** Comparison of general and disease characteristics of subjects in the two groups.

	Mucus plug group (*n* = 95)	Control group (*n* = 56)	*P-*value
Age (years)	6.83 [4.67–9.17]	6.13 [4.17–9.08]	0.316
Male	57 (60.0%)	31 (55.4%)	0.573
Urban living	71 (74.7%)	44 (78.6%)	0.593
Birth weight (kg)	3.30 [3.00–3.50]	3.25 [3.00–3.60]	0.997
Premature	4 (4.4%)	5 (9.3%)	0.220
Cesarean	42 (44.7%)	31 (57.4%)	0.136
Neonatal asphyxia	0 (0.0%)	1 (1.9%)	–
Neonatal oxygen	1 (1.1%)	1 (1.9%)	–
Feeding mode			0.174
Breastfeeding	71 (75.5%)	40 (72.7%)	
Formula feeding	13 (13.8%)	4 (7.3%)	
Mixed feeding	10 (10.6%)	11 (20.0%)	
Allergy history	37 (38.9%)	26 (46.4%)	0.368
Allergic family history	39 (41.5%)	22 (40.0%)	0.858
Siblings ≥1	38 (40.0%)	20 (35.7%)	0.601
Asthma hospitalization history	24 (25.8%)	8 (14.3%)	0.097
Systemic glucocorticoid therapy	13 (14.0%)	4 (7.3%)	0.216
Admission season			0.935
Spring	18 (18.9%)	11 (19.6%)	
Summer	21 (22.1%)	10 (17.9%)	
Autumn	41 (43.2%)	25 (44.6%)	
Winter	15 (15.8%)	10 (17.9%)	
Emergency admission	51 (53.7%)	27 (48.2%)	0.516
PICU treatment	19 (20.0%)	7 (12.5%)	0.238
First asthma diagnoses	83 (87.4%)	43 (76.8%)	0.091
Respiratory infection	87 (91.6%)	46 (82.1%)	0.084
Comorbidity	49 (51.6%)	23 (41.1%)	0.212
Asthma phenotype			0.614
Atopy-only	47 (59.5%)	26 (56.5%)	
Eos-only	5 (6.3%)	1 (2.2%)	
T2-high	15 (19.0%)	9 (19.6%)	
T2-low	12 (15.2%)	10 (21.7%)	
Severe to critical attacks	26 (27.4%)	19 (33.9%)	0.395
Dyspnea	50 (52.6%)	15 (26.8%)	**0**.**002**
Respiratory failure	13 (13.7%)	9 (16.1%)	0.688
Serum total IgE (IU/mL)	286.85 [119.00–484.90]	396.00 [248.18–1,265.00]	**0**.**027**
Positive serum allergen detection	71 (74.7%)	40 (71.4%)	0.318
Procalcitonin	0.15 [0.08–0.46]	0.22 [0.09–0.53]	0.758
C-reactive protein	11.00 [3.35–22.00]	8.00 [3.43–12.80]	0.161
White blood cell counts	9.03 [6.81–11.86]	8.16 [7.02–11.15]	0.465
Neutrophil count	5.49 [3.49–8.24]	5.40 [3.55–6.83]	0.460
Eosinophil count	0.12 [0.01–0.35]	0.21 [0.02–0.41]	0.147
Eosinophil %	1.10 [0.10–4.50]	2.55 [0.30–4.50]	0.154

The bold values indicated that *P* < 0.05.

Compared to the control group, subjects with dyspnea had a higher proportion in the mucus plug group (52.6% vs. 26.8%, *P* < 0.01). The serum total IgE level of the mucus plug group was lower than the control group (286.85 IU/mL vs. 396.00 IU/mL, *P* < 0.05). The proportion of subjects who diagnosed asthma for the first time (87.4% vs. 76.8%, *P* > 0.05) and combined with respiratory infection including pneumonia and bronchitis (91.6% vs. 82.1%, *P* > 0.05) in the mucus plug group were both higher than that in the control group. Our study exhibited a higher proportion of subjects admitted to the hospital during autumn compared to other seasons. Still, the distribution of subjects was not found to be different in the two groups. No significant differences were detected between the two groups in admission mode, comorbidity, asthma phenotype, severity, positive serum allergen detection, or respiratory failure (*P* > 0.05). Table 1 also revealed that there were no differences in the results of procalcitonin, C-reactive protein, white blood cell count, neutrophil count, eosinophil count, and eosinophil percentage among the three groups (*P* > 0.05).

Compared to the control group, more subjects were administered systemic glucocorticoid (Methylprednisolone: 43.2% vs. 28.6%), aminophylline (2.1% vs. 0.0%), and mucolytic drug (Ambroxol or Bromhexine: 68.4% vs. 64.3%) after hospitalization in the mucus plug group, but it was not statistically significant to say that these differ. The proportion of subjects who needed respiratory support had no difference between the two groups (χ^2^ = 4.225, *P* = 0.121). Additionally, none of the subjects in the control group received intravenous magnesium sulfate. The proportion of subjects who received intravenous magnesium sulfate was 7.4% in the mucus plug group, which was significantly higher than that in the control group (*P* = 0.047).

### 4.3 Analysis of relative factors of mucus plug in asthma children

In Logistic regression analyses, the dependent variable was the result of mucus plugs under the bronchoscope (with mucus plugs = 1, without mucus plugs = 0). Since eosinophil count and eosinophil percentage were correlated, we could only include eosinophil percentage in the multivariable analysis to prevent collinearity (as shown in Supplementary Table S5). As shown in Table 2, the results showed that only presenting dyspnea signs at admission [OR = 3.037 (1.485–6.212), *P* = 0.002] was associated with mucus plug in children hospitalized for an acute asthma attack, in univariable analysis. In multivariable analysis, first asthma diagnoses during hospitalization [OR = 4.404 (1.101–17.614), *P* = 0.036], dyspnea [OR = 4.039 (1.306–12.496), *P* = 0.015], and cesarean [OR = 0.274 (0.092–0.812), *P* = 0.019] might be associated with mucus plugs in children hospitalized for an acute asthma attack.

**Table 2:** Factors associated with mucus plugs in children hospitalized for asthma attack.

	*β*	Standard error	Odds ratio [95% CI]	*P*-value
First asthma diagnoses	1.483	0.707	4.404 [1.101–17.614]	**0**.**036**
Dyspnea	1.396	0.576	4.039 [1.306–12.496]	**0**.**015**
Cesarean	1.296	0.555	0.274 [0.092–0.812]	**0**.**019**
Eosinophil percentage	−0.158	0.084	0.854 [0.724–1.006]	0.059
Constant	−1.578	0.793	0.206	**0**.**047**

The bold values indicated that *P* < 0.05.

## 5 Discussion

Mucus plugs can persist in the bronchopulmonary segment of asthmatic airways for extended periods, exerting long-lasting and persistent effects on patients with asthma (7). In our study, the mucus plugs were assessed through the result of bronchoalveolar lavage. Subjects were categorized into the mucus plug group and the control group. In our study, 62.9% of the subjects admitted to the hospital with acute asthma attacks and underwent bronchoscopy had mucus plugs. This rate is comparable to the findings of Dunican EM et al. (8). The frequency of airway mucus plugs might vary in different levels of asthma control. One study found that all asthma children with respiratory failure treated in the ICU had mucus plugs (9). In contrast, our study investigated subjects hospitalized for an acute asthma attack, including those treated in general wards. We found that 14.6% (22/151) of subjects experienced respiratory failure, and only 13.7% in the mucus plug group. According to the diagnostic criteria for children's respiratory failure, the figures were dependable, and can accurately reflect the current situation of our hospital. The requirement for O2, oxygen saturation, and SpO2 were crucial in clinical practice. However, the determination of respiratory failure cannot be made based on this information in the clinical setting. Meanwhile, the analysis comparing the mucus plug group to the control group revealed that more subjects presented with dyspnea symptoms on admission were hospitalized for their initial asthma diagnosis, and received intravenous magnesium sulfate. In the multivariable analysis, the results indicated that the first diagnosis of asthma and dyspnea were the relative factors for mucus plugs in children with asthma.

Some studies suggested that airway mucus plug was associated with eosinophilic inflammation (10, 11). Inflammatory factors related to eosinophilic inflammation may stimulate airway epithelial goblet cell proliferation and an increase in mucin 5AC expression (12, 13). Eosinophils in airway secretions may impede airway mucin decomposition, raise mucus viscosity, impair mucus cilia transport function, and foster airway mucus plug formation (14). Our study did not find notable variance in asthma phenotype distribution between the mucus plug group and the control group. The retrospective design might contribute to this observation. The available data on eosinophils and specific IgE in peripheral blood was limited in our study. The OR value of serum total IgE equaled 1.00, indicating that serum total IgE did not associate with the mucus plugs in our study. In logistic analysis, no association between eosinophils (count and percentage) and mucus plugs was found in this study. Glucocorticoid therapy has significant effects on eosinophil count and percentage (15, 16), which needs to be further explored in multi-center prospective clinical research.

Though we found the proportion of subjects with comorbidity in the mucus group was higher than that in the control group (51.6% vs. 41.1%), there was no statistical significance found at this stage. The mechanism of type 2 inflammation suggests that various type 2 inflammatory diseases frequently coexist, emphasizing the significance of evaluating and treating comorbidity in asthma diagnosis and management (17). The presence of comorbidity correlated with type 2 inflammation in pediatric asthma patients. Airway epithelial barrier destruction diminishes the ability of airway cilia to clear mucus and contributes to airway mucus plugs. The complex interaction between injury to the epithelial barrier and type 2 immunity contributes to type 2 inflammation (18). During type 2 inflammation, Th2 cells generate type 2 cytokines that may lead to the impairment of the barrier function. Additionally, these cytokines can prompt the release of alarms such as thymic stromal lymph protein (TSLP), interleukin (IL)-33, and IL-25 by epithelial cells, activate type II innate lymphoid cell (ILC2), secrete more type 2 cytokines, and act on effector cells such as eosinophils. These actions may result in elevated production of inflammatory mediators and chemokines, leading to the development of a vicious cycle of type 2 inflammation and ultimately increasing the risk of mucus plugs (19).

In our study, according to the OR value, cesarean delivery might also be associated with the formation of mucus plugs in asthma children. The hygienic hypothesis suggested that reducing microbial exposure in early life may impact the development of children's flora, disrupt the balance between Th1 and Th2 immunity, and increase the likelihood of allergic diseases (20). Moreover, the formation of mucus plugs is subject to a multitude of factors. At this juncture, the significance of cesarean delivery may be discernible only in statistical terms, and the clinical value remains unclear. In other research, the types of airway inflammation in asthma can have a significant relationship with the mucus plugs (11). However, this study found no association between Th1 and Th2 markers and mucus plug formation. As mentioned above, this might be influenced by glucocorticoid therapy before admission. The relationship between cesarean delivery, airway inflammation, and mucus plugs represents an intriguing area of investigation that warrants further analysis.

Our study had some limitations. First, it should be noted that this study was retrospective, which may have introduced a degree of sampling bias. Bronchoscopy is rarely undertaken in children hospitalized with asthma and is indeed contraindicated in general. To enhance the practicality of this approach in a clinical setting, we are currently investigating the potential for identifying asthma signs in children undergoing bronchoscopy. Given the grade of the hospital, there may be a bias of disease severity in children with asthma who were hospitalized for treatment. This could result in a higher rate of mucus plugs among children with asthma who need hospitalization. Additionally, retrospective research methods resulted in incomplete or unavailable clinical information and laboratory test results for individual children. At that moment we could only collect seldom patients' cell fractionation results of bronchoalveolar lavage fluid. We did not describe it because these results were not representative. Furthermore, due to the constraints imposed by retrospective studies, the data on adverse events of undergoing bronchoscopy was neither inadequate to represent this group. Limited by the evaluation method of airway mucus plugs, the study focused on children who undergo bronchoscopy, which may have selection bias. Though the mucus plugs were the most common reason for asthma children to undergo the bronchoscopy examination, the patients who performed a bronchoscopy examination were based on the physician's professional opinion. When comparing the general and disease characteristics of subjects who underwent bronchoscopy examination to those who did not, we only found significant differences in hospitalization for asthma within the past 12 months, admission to the hospital through the emergency department, and requirement for treatment in the PICU. The details for the bronchoscopy report to classify the mucus plug were clear and had been reported before (21). Based on the assessment method, this study could not determine the location (airway size/depth or lobe) of the mucus plugs. This is one of the limitations of our study. Furthermore, we conducted another research using Multi-Detector-Row Computed Tomography to address this question. In the research about adults with chronic obstructive pulmonary disease, 30% of individuals with CT evidence of mucus plugging had no mucus-related symptoms (22). The CT scan, with the advantage of being less invasive, might also provide a unique finding for mucus plugs in children with asthma. Finally, some common objective indicators before enrollment (ACT, c-ACT, etc.) and treatment before hospitalization in subjects were unattainable. Anyway, the widespread adoption of EMRS has made observational research, which can provide clinical insights, more feasible. Our research was conducted using a clinic-based data source, which could ensure the reliability of our findings.

## 6 Suggests

In clinical practice, it is essential to identify subjects at risk and implement appropriate interventions to prevent the formation of mucus plugs and minimize their impact on asthma management. Our study revealed associated factors for asthma mucus plugs, including those hospitalized for their initial asthma diagnosis and presenting with dyspnea signs on admission. While our study is retrospective, further research is needed for exploration.

## Data Availability

The original contributions presented in the study are included in the article/Supplementary Material, further inquiries can be directed to the corresponding author.
