# Supplementary material for: Clinical characteristics and factors associated with mucus plugs under bronchoscopy in children hospitalized for acute asthma attack
Source: Front Pediatr. 2024 Oct 14;12:1382680. doi: 10.3389/fped.2024.1382680 (PMC11513321; doi:10.3389/fped.2024.1382680)
Supplement: Supplementary file 1 [file Datasheet1.pdf]

Supplementary Table 1 Assessment of Acute Asthma Attacks in Children Aged 6 and Above

	Mild	Moderate	Severe	Critical
Breathlessness	Walking	Speaking	At rest	Dyspnea
Position	Lie on the back	Sit	kyphotic	unsteady
Speaks in	Sentences	Phrases	Words	Unable to speak
Consciousness	Have anxiety, irritability	Often anxious and irritable	Often anxious and irritable	Drowsiness, confusion
Accessory muscle use	No	Common	Marked	Paradoxical
Wheeze	Variable	Moderate to loud	Loud-on both inhalation and exhalation	Often quiet
Pulse	Slight increase	Increase	Slight increase	Slow or irregular
PEF (% of predicted or personal best)	After SABA ＞80	Before SABA: 50-80; After SABA:60-80	Before SABA⩽50 After SABA⩽60	Unable to complete
SaO2 (% on air)	0.90-0.94	0.90-0.94	0.90	＜0.90

When assessing the severity of an acute attack, as long as there is an index of a certain severity, it can be classified into this severity level. PEF: Maximum peak expiratory flow; SABA: short-acting β2 receptor agonist

Supplementary Table 2 Assessment of Acute Asthma Attacks in Children Under 6 Years

	Mild	Severe
Consciousness	No	Anxiety, irritability, lethargy, or confusion
SaO2 (% on air)	⩾0.92	＜0.92
Speaks in	Sentences	Words
Pulse	＜100	＞200(0-3years); ＞180(4-5years)
Cyanosis	No	Might exist
Wheeze	Variable	Often quiet

When assessing the severity of an acute attack, as long as there is an index of a certain severity, it can be classified into this severity level.

Supplementary Table 3 Comparison of general characteristics of subjects with and without bronchoscopy examination

	Performed bronchoscopy group (n=151)	Not performed bronchoscopy group (n=91)	*P* value
Age (years)	6.67 [4.58-9.17]	5.42 [4.00-9.75]	0.298
Male	88(58.3%)	55(60.4%)	0.740
Urban living	115(76.2%)	73(80.2%)	0.462
Birth weight (kg)	3.30 [3.00-3.50]	3.30 [3.00-3.70]	0.306
Premature	9(6.1%)	5(5.5%)	0.851
Cesarean	73(49.3%)	42(46.2%)	0.634
Neonatal asphyxia	1(0.7%)	0(0.0%)	-
Neonatal oxygen	2(1.4%)	2(2.2%)	-
Feeding mode			0.274
Breastfeeding	111(74.5%)	57(64.8%)	
Formula feeding	17(11.4%)	13(14.8%)	
Mixed feeding	21(14.1%)	18(20.5%)	
Allergy history	63(41.7%)	28(31.1%)	0.100
Allergic family history	61(40.9%)	46(50.5%)	0.146
Siblings≥1	58(38.4%)	38(41.8%)	0.606
Asthma hospitalization history	32(21.5%)	7(7.7%)	**0.005**
Systemic glucocorticoid therapy	17(11.5%)	4(4.5%)	0.216

Supplementary Table 4 Comparison of disease characteristics of subjects with and without bronchoscopy examination

	Performed bronchoscopy group (n=151)	Not performed bronchoscopy group (n=91)	*P* value
Admission season			0.131
Spring	29(19.2%)	16(17.6%)	
Summer	31(20.5%)	23(25.3%)	
Autumn	66(43.7%)	46(50.5%)	
Winter	25(16.6%)	6(6.6%)	
Emergency admission	78(51.7%)	69(75.8%)	**0.000**
PICU treatment	26(17.2%)	24(26.4%)	**0.021**
First asthma diagnoses	126(83.4%)	74(81.3%)	0.672
Respiratory infection	133(88.1%)	80(87.9%)	0.969
Comorbidity	72(47.7%)	53(58.2%)	0.111
Asthma phenotype			0.203
Atopy-only	73(58.4%)	56(71.8%)	
Eos-only	6(4.8%)	1(1.3%)	
T2-high	24(19.2%)	10(12.8%)	
T2-low	22(17.6%)	11(14.1%)	
Severe to critical attacks	45(29.8%)	34(37.4%)	0.224
Dyspnea	65(43.0%)	45(49.5%)	0.332
Respiratory failure	22(14.6%)	20(22.0%)	0.140
Serum total IgE (IU/mL)	334.24 [132.00-700.00]	257.00 [99.70-637.87]	0.092
Positive serum allergen detection	111(86.0%)	69(88.5%)	0.617
Procalcitonin	0.16 [0.09-0.50]	0.09 [0.05-0.19]	**0.002**
C-reactive protein	8.56 [3.43-19.90]	8.00 [3.00-15.00]	0.302
White blood cell counts	8.88 [7.00-11.69]	9.94 [7.81-13.17]	**0.042**
Neutrophil count	5.41 [3.49-7.96]	6.99 [4.66-9.53]	**0.011**
Eosinophil count	0.14 [0.02-0.38]	0.05 [0.01-0.25]	**0.013**
Eosinophil %	1.40 [0.20-4.50]	0.40 [0.10-2.00]	**0.001**

Supplementary Table 5 The collinearity diagnostics of variable assignment in regression analysis

	Tolerance	Variance inflation factor
Asthma hospitalization history	0.897	1.115
First asthma diagnoses	0.898	1.114
Respiratory infection	0.887	1.140
Dyspnea	0.850	1.176
Cesarean	0.900	1.111
C-reactive protein	0.884	1.132
Eosinophil count	0.106	9.413
Eosinophil percentage	0.108	9.225
Serum total IgE (IU/mL)	0.869	1.150

If the tolerance (Tol) is less than 0.1 or the variance inflation factor (VIF) is greater than 10, collinearity is present. When VIF > 5.0, it should question the presence of collinearity.
